# A memory-improving dipeptide, Tyr-Pro, can reach the mouse brain after oral administration

**DOI:** 10.1038/s41598-023-44161-z

**Published:** 2023-10-07

**Authors:** Lihong Cheng, Mitsuru Tanaka, Atsuko Yoshino, Yuki Nagasato, Fuyuko Takata, Shinya Dohgu, Toshiro Matsui

**Affiliations:** 1 Department of Bioscience and Biotechnology, Faculty of Agriculture, Graduate School of Kyushu University, 744 Motooka, Nishi-Ku, Fukuoka, 819-0395 Japan; 2 Department of Pharmaceutical Care and Health Sciences, Faculty of Pharmaceutical Sciences, Fukuoka University, Fukuoka, Japan

**Keywords:** Nutritional supplements, Pharmacodynamics

## Abstract

The transport and accumulation of orally administered functional food-derived peptides in the brain was not fully explored. Thus, in the present study, we aimed to provide critical evidence regarding brain accumulation of a memory-improving soy dipeptide, Tyr-Pro, following oral administration. Stable isotope-labeled Tyr-Pro (Tyr-[^13^C_5_,^15^N]Pro) was orally administered to male ICR mice at 10 or 100 mg/kg. Surprisingly, the intact labeled Tyr-Pro exhibited maximal plasma and brain levels 15 min after administration (plasma: area under the curve [*AUC*_0–120 min_], 1331 ± 267 pmol·min/mL-plasma; brain: *AUC*_0–120 min_ of 0.34 ± 0.11 pmol·min/mg-dry brain, at 10 mg/kg). In addition, we detected labeled Tyr-Pro in the brain parenchyma, indicating a validated blood–brain-barrier (BBB) transportability. Moreover, we confirmed the preferable accumulation of Tyr-Pro in the hypothalamus, hippocampus, and cortex with > 0.02 pmol/mg-tissue. In conclusion, we provided the first evidence that orally administered Tyr-Pro at 10 mg/kg directly entered the blood circulation with an absorption ratio of 0.15%, of which 2.5% of Tyr-Pro was transported from the plasma to the mouse brain parenchyma.

## Introduction

Cognitive-related diseases such as Alzheimer's disease (AD) are well-known to markedly impair memory and negatively affect daily activities. According to the World Health Organization (WHO), an estimated 50 million individuals worldwide suffer from dementia, which is expected to more than triple by 2050^1^. However, to date, optimal therapeutic strategies are yet to be established. In recent decades, there has been a growing interest in the research and development of foods with health-promoting effects, also known as functional foods, which contain different types of molecules with diverse physiological functions, including vitamins, polyphenols, lipids, and peptides^2^. Among them, peptides are one of the most important and widely studied functional food-derived materials, reportedly exhibiting various physiological effects, including beneficial effects on the brain^3–6^. Before establishing their potential effects, it is essential to determine whether candidate compounds can reach their target brain regions across the blood–brain barrier (BBB) system, as well as elucidate the mechanism underlying physiological responses^7^.

The BBB strictly regulates the transport of substances into the brain. In the past few decades, several methods, including in silico, in vitro, in situ, and in vivo models, have been established to examine the transport of molecules across the BBB^8^. KS-487 was discovered as an LDL receptor-related protein 1 (LRP1)-binding 15 mer cyclic peptide and its permeability has been demonstrated using in vitro rat and monkey BBB kit models^9^. However, a major limitation of currently available in vitro models is that they fail to comprehensively mimic the native BBB, given the use of plastic and porous transwell membrane inserts, thus affording inconsistent results when compared with those of in vivo experiments^10,11^. An in vivo evaluation of BBB permeation has been performed by liquid chromatography (LC)-mass spectrometry (MS), using homogenized or microdialyzed brain tissues after perfusion experiments^12^, or by visualization analysis using positron-emission tomography (PET)^12^ and mass spectrometric (MS) imaging techniques^13^. Notably, the invasiveness of microdialysis may disrupt the BBB system, resulting in poorly reliable observations^14^. In addition, magnetic resonance imaging, quantitative radioautography, fluorescence, and PET visualization techniques have limitations in terms of preparing fluorophore- or radio-isotope-labeled target small molecules without altering physicochemical properties^15^. Hence, qualitative and quantitative assessments of the BBB permeability of target molecules remain technically challenging. To date, the oral administered memory-improved peptide, Gly-Thr-Trp-[carboxyl-^14^C]Tyr, was shown to be distributed in mouse brain tissue using the radioactive tracing technique^16^. However, it may be difficult to distinguish between target peptides and fragmented (metabolized) peptides.

Previously, we had successfully demonstrated that Gly-Sar, Gly-Pro, and Tyr-Pro were intact BBB transportable dipeptides as determined by conducting in situ mouse brain perfusion experiments. Furthermore, matrix-assisted laser desorption ionization (MALDI)-MS imaging was used to demonstrate an interesting finding regarding brain tissue accumulation, wherein Tyr-Pro was confirmed to be accumulated in the parenchyma of the mouse brain, particularly in the hippocampus, cerebral cortex, cerebellum, hypothalamus, and striatum regions^17^. In a long-term oral administration study assessing Tyr-Pro in amyloid β-injected AD model mice, we provided the first evidence that orally administered Tyr-Pro can improve impaired cognitive deficits, considering working and long-term memory^18^. These in situ and in vivo findings indicate that Tyr-Pro could be developed as an orally effective functional food in preventing cognitive impairment. Irrespective of the preference for Tyr-Pro intake, the bioavailability remains elusive, including the entry into the circulating blood system and the brain without degradation.

**Figure 1:**
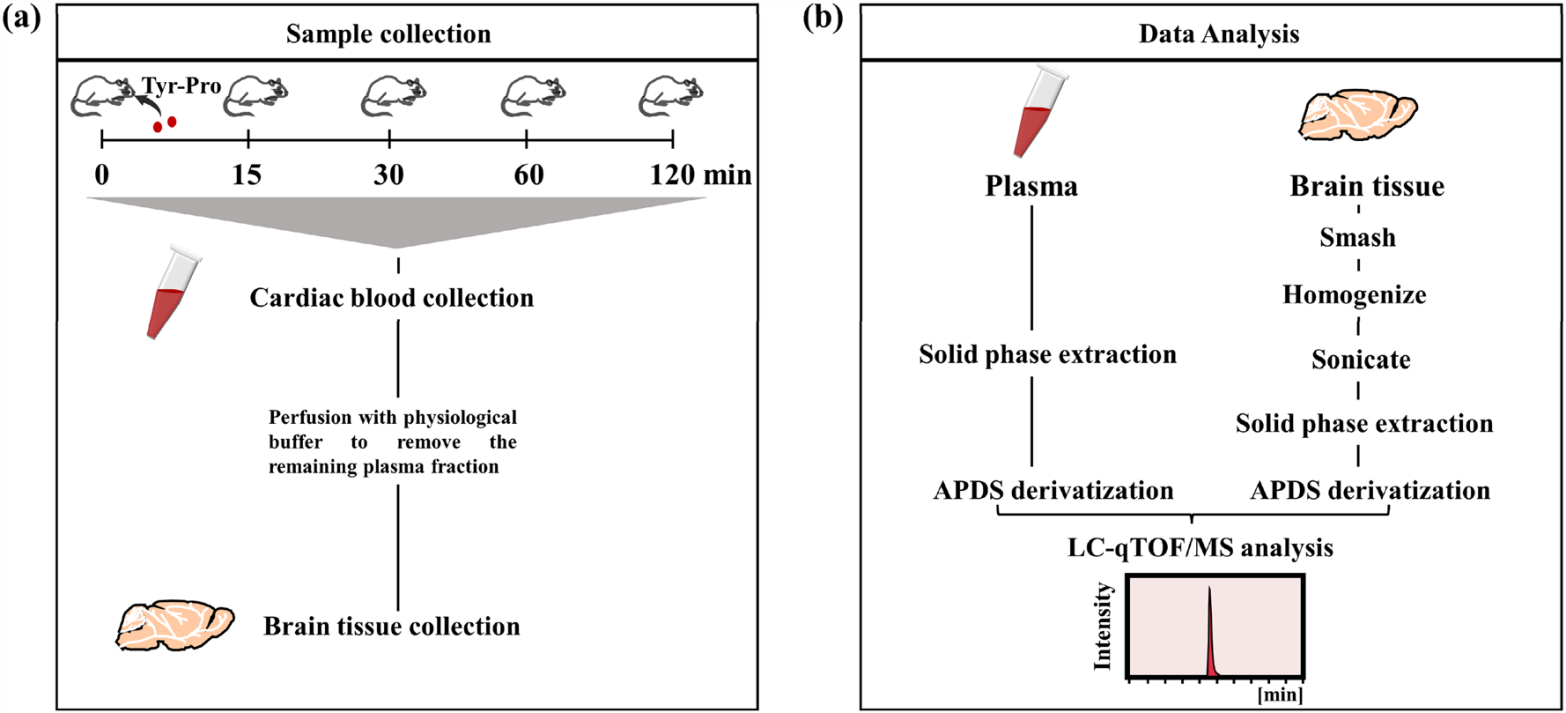
Schematic overview of animal experiments and detection workflow. (**a**) Graphic illustration of the workflow to acquire mouse plasma and brain tissue. The ICR mice were orally administrated 10 and 100 mg/kg Tyr-[^13^C_5_,^15^N]Pro, and then the plasma and brain tissue were collected at 0, 15, 30, 60, and 120 min. (**b**) Flow chart clarifying the plasma and brain tissue processing protocol. Plasma and brain tissue were applied to a solid phase extraction, reacted with APDS, and then analyzed by LC-qTOF/MS. APDS, 3-aminopyridyl-*N*-hydroxysuccinimidyl carbamate; LC-qTOF/MS, liquid chromatography-quadrupole time-of-flight/mass spectroscopy.

In the present study, we aimed to determine whether Tyr-Pro can reach the brain parenchyma in an intact form after oral administration to mice, according to the protocol shown in Fig. 1. To confirm the intact accumulation of Tyr-Pro, highly sensitive detection of Tyr-Pro using LC-quadrupole time-of-flight (qTOF)/MS was established using a 3-aminopyridyl-*N*-hydroxysuccinimidyl carbamate (APDS) derivatization technique^19^.

## Results

### APDS derivatization-LC-qTOF/MS performance of Tyr-Pro detection

**Figure 2:**
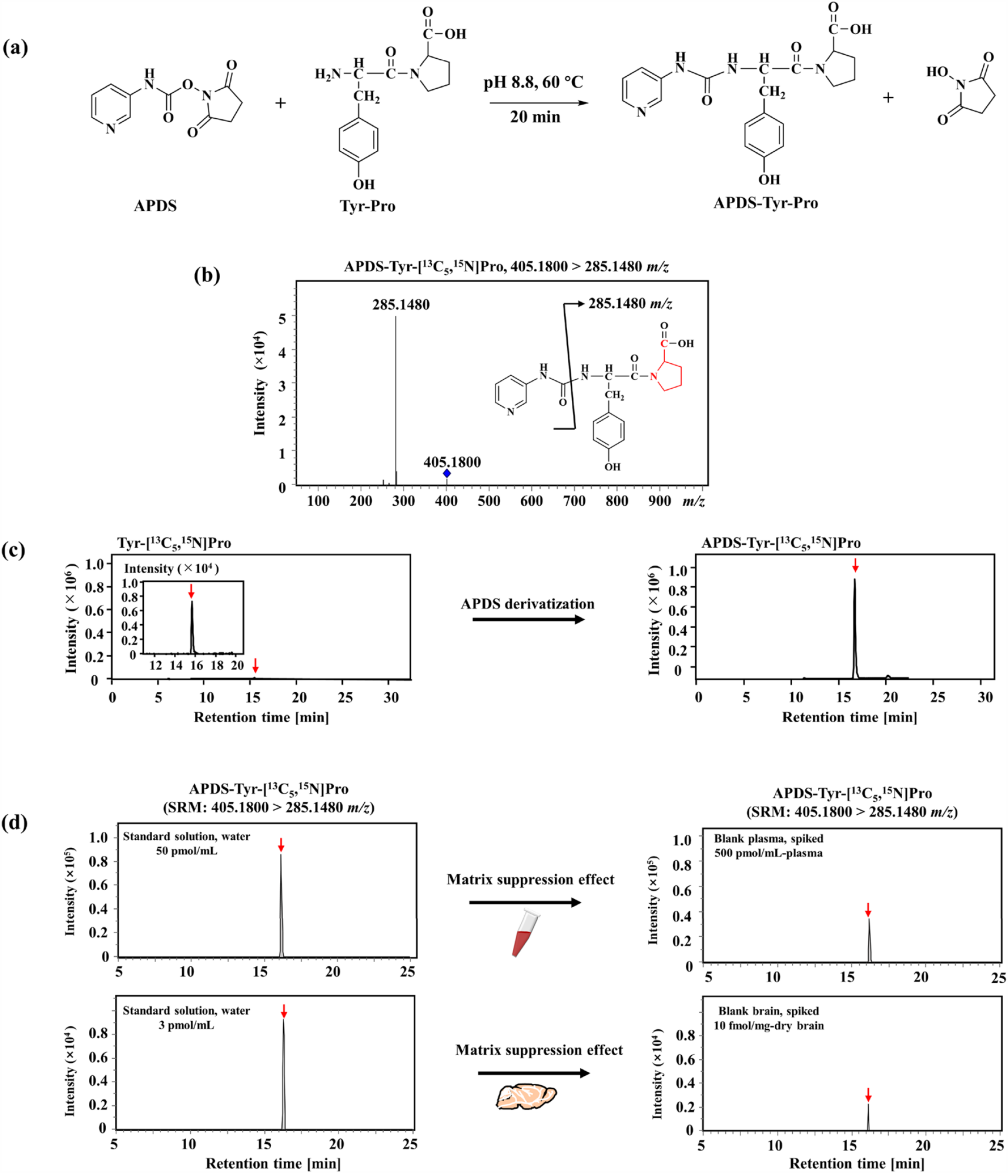
APDS derivatization-LC-qTOF/MS performance of Tyr-Pro. (**a**) Scheme of APDS derivatization reaction of Tyr-Pro. APDS derivatization conditions are described in the Materials and method section. (**b**) MS/MS infusion analyses of APDS-Tyr-[^13^C_5_,^15^N]Pro. Isotope-labeled atoms are highlighted in red on the inserted structure. (**c**) A highly sensitive dipeptide detection method based on APDS derivatization LC-qTOF/MS. Extracted ion chromatograms of intact Tyr-[^13^C_5_,^15^N]Pro (285.1480 ± 0.005 *m/z*) and APDS-derivatized Tyr-[^13^C_5_,^15^N]Pro (405.1800 > 285.1480 *m/z*) by SRM-LC-qTOF/MS analysis. A red arrow indicates the peak of the target molecule. (**d**) Matrix suppression effect on the detection of APDS-Tyr-[^13^C_5_,^15^N]Pro in mouse plasma and brain homogenate. The same concentration of Tyr-[^13^C_5_,^15^N]Pro was spiked either in standard solution (water) or blank plasma and blank brain homogenate under the optimized SRM-LC-qTOF/MS condition. A red arrow indicates the peak of the target molecule.

To exclude the influence of endogenous Tyr-Pro^20^, mice were orally administered a stable isotope-labeled Tyr-[^13^C_5_,^15^N]Pro. [^13^C_9_,^15^N]Tyr-Pro was used as the internal standard (IS) for LC-qTOF/MS measurements. APDS was employed as an amine derivatization reagent, given its expected high ionization efficiency of derivatives in LC–MS/MS^19^. In the current study, APDS-amine derivatization of the two isotope-labeled peptides was performed with 300 mM APDS at 60 °C and pH 8.8 for 20 min (Fig. 2a). Considering this reaction, the observed product was a monoisotopic precursor ion at 405.1800 *m/z* ([M + H]^+^) for Tyr-[^13^C_5_,^15^N]Pro attached to the APDS moiety (+ 120 Da) ([^13^C_9_,^15^N]Tyr-Pro: [M + H]^+^, 409.1920 *m/z*). For the quantitative and selective detection of the two labeled dipeptides, it is essential to confirm whether APDS derivatization is applicable for LC–MS detection of target dipeptides in plasma and brain samples without any interference with the sample matrix. Accordingly, we measured the two labeled Tyr-Pro targets with the help of selected reaction monitoring (SRM) analysis under the monitoring ions of Tyr-[^13^C_5_,^15^N]Pro (405.1800 > 285.1480 *m/z*) and [^13^C_9_,^15^N]Tyr-Pro (409.1920 > 289.1600 *m/z*) (Fig. 2b and Table S1). Based on the SRM analysis, we observed a highly selective (or non-interfering) MS peak for APDS-Tyr-[^13^C_5_,^15^N]Pro, along with the marked enhancement of MS detection using APDS derivatization (by a factor of approximately 150), compared with the detection of non-derivatized Tyr-[^13^C_5_,^15^N]Pro (Fig. 2c). Figure 2d shows the matrix effect on the detection of APDS-Tyr-[^13^C_5_,^15^N]Pro in mouse plasma and brain homogenate spiked at a concentration of 500 pmol/mL-plasma or 10 fmol/mg-dry brain. Compared with the MS intensity of APDS-Tyr-[^13^C_5_,^15^N]Pro in aqueous solution, a 62% and 76% reduction in MS intensity was observed owing to the matrix effect in plasma and brain homogenate, respectively. Thus, the quantification of Tyr-[^13^C_5_,^15^N]Pro by APDS-aided SRM-LC-qTOF/MS analysis was performed according to calibration curves individually prepared for plasma and brain samples.

In the SRM-LC-qTOF/MS performance for a targeting dipeptide, Tyr-Pro, we obtained good calibration curves for APDS-Tyr-[^13^C_5_,^15^N]Pro in both plasma and brain samples, aided by the addition of [^13^C_9_,^15^N]Tyr-Pro as an IS (100 pmol/mL-plasma or 0.1 pmol/mg-dry brain). The calibration curve for plasma was calculated as y = 0.0295x + 0.0101 (10–1500 pmol/mL-plasma, R^2^ = 0.9891), where y is the peak area ratio (observed peak area of the peptide against that of IS) and x is the peptide concentration (pmol/mL-plasma). Considering brain samples, the calibration curve was obtained as y = 37.363x + 0.0706 (0.005–0.075 pmol/mg-dry brain, R^2^ = 0.9954), where y is the peak area ratio against the IS and x is the peptide concentration in dry brain tissue (pmol/mg-dry brain). Based on the current SRM-LC-qTOF/MS, if at least 6 fmol/mg-dry brain of Tyr-[^13^C_5_,^15^N]Pro was achieved in brain tissue, the presence of the target can be significantly detected, based on a limit of detection (LOD) of 9.99 pmol/mL-plasma and 0.006 pmol/mg-dry brain, in plasma and brain, respectively, as well as a limit of quantitation (LOQ) of 30.3 pmol/mL-plasma and 0.019 pmol/mg-dry brain, in plasma and brain, respectively (Table S2). In addition, the aforementioned good linearity of both calibration curves obtained using the IS strongly indicated that the 300 mM APDS concentration, as employed in the present study, was sufficient for the appropriate derivatization of target dipeptides, even if amine contaminants from plasma and sample matrixes may affect the APDS derivatization efficiency.

### Absorption of Tyr-Pro into the blood system after oral administration in ICR mice

**Figure 3:**
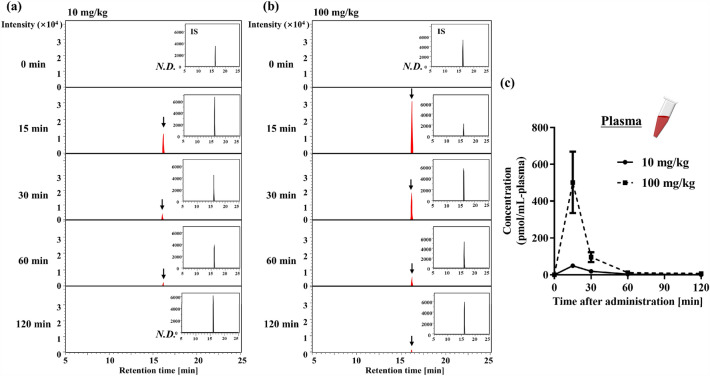
Absorption of Tyr-Pro into the plasma after oral administration to ICR mice. (**a**, **b**) SRM chromatograms of LC–MS/MS analysis of APDS-Tyr-[^13^C_5_,^15^N]Pro into the blood after single oral administration to mice. Representative SRM chromatograms of APDS-Tyr-[^13^C_5_,^15^N]Pro (405.1800 > 285.1480 *m/z*) in the plasma after single oral administration of 10 and 100 mg/kg for different time points (0–120 min) were shown. The corresponding chromatograms of internal standard (IS) of [^13^C_9_,^15^N]Tyr-Pro (409.1920 > 289.1800 *m/z*) were inserted as a small window for each chromatogram. The peak of the target molecule is indicated by an arrow. *N.D.* indicates not detected. The plasma sample was subjected to an APDS derivatization, followed by LC–MS/MS-SRM analysis as described in the Materials and method section. (**c**) Time course of plasma concentrations of Tyr-[^13^C_5_,^15^N]Pro after single oral administration at doses of 10 (solid line) and 100 mg/kg (dotted line) in mice. The values of each time point at different doses were calculated using the present plasma calibration curve. Each value is expressed as mean ± SEM (*n* = 3–5).

Following oral administration of Tyr-[^13^C_5_,^15^N]Pro (10 and 100 mg/kg, corresponding to 1 and 11 μmol/mouse, respectively), cardiac blood sampling was performed to establish the absorption behavior in the circulating blood system. APDS-aided LC-qTOF/MS analysis of plasma samples obtained from individual mice at 15, 30, 60, and 120 min revealed the presence of intact Tyr-[^13^C_5_,^15^N]Pro in plasma at any time post-administration (Fig. 3a and b). With the help of [^13^C_9_,^15^N]Tyr-Pro as an IS, the absorption kinetics of Tyr-[^13^C_5_,^15^N]Pro in mouse plasma were evaluated as *C*_max_ values of 49 ± 21 and 502 ± 167 pmol/mL-plasma, with* t*_max_ of 15 min, *t*_1/2_ of 12 and 8 min, and area under the curve (*AUC*_0–120 min_) of 1331 ± 267 and 10,424 ± 4066 pmol·min/mL-plasma at 10 and 100 mg/kg, respectively (Fig. 3c and Table 1). In ICR mice, orally administered Tyr-[^13^C_5_,^15^N]Pro at 10 and 100 mg/kg was absorbed intact across the intestinal membrane, with an absorption ratio of 0.15 ± 0.03% (1.46 ± 0.29 nmol/whole plasma) and 0.10 ± 0.04% (11.5 ± 4.5 nmol/whole plasma), respectively.

**Table 1:** Pharmacokinetic parameters of Tyr-[^13^C_5_,^15^N]Pro in the plasma after oral administration to ICR mice at 10 and 100 mg/kg.

Parameter	Dose (mg/kg)	
	10	100
*C*_max_ (pmol/mL-plasma)	49 ± 21	502 ± 167
*t*_max_ (min)	15	15
*t*_1/2_ (min)	12	8
*AUC*_0–120 min_ (pmol·min/mL-plasma)	1331 ± 267	10,424 ± 4066

*C*_max_ maximum plasma concentration, *AUC*_0–120 min_ area under the plasma concentration, *t*_max_ time to reach peak plasma concentration, *t*_1/2_ elimination half-life.

Each value is expressed as mean ± SEM (*n* = 3–5).

### Tyr-Pro entry into the brain after single oral administration in ICR mice

**Figure 4:**
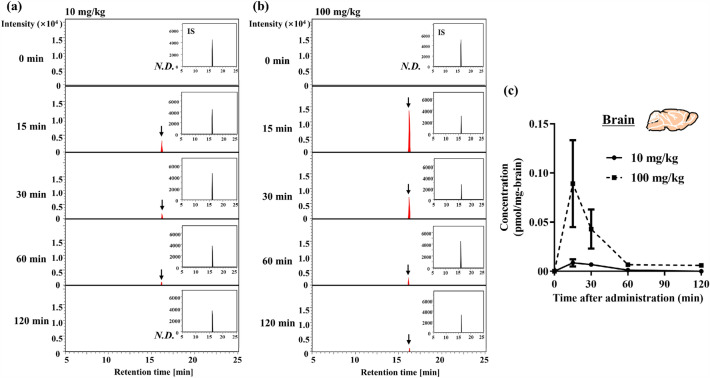
Entry of Tyr-Pro into the brain after single oral administration to ICR mice. (**a**, **b**) SRM chromatograms of LC–MS/MS analysis of APDS-Tyr-[^13^C_5_,^15^N]Pro in brain tissue after single oral administration to mice. Representative SRM chromatograms of APDS-Tyr-[^13^C_5_,^15^N]Pro (405.1800 > 285.1480 *m/z*) in the brain tissue after oral administration of 10 and 100 mg/kg for different time points (0–120 min) were shown. The corresponding chromatograms of internal standard (IS) of [^13^C_9_,^15^N]Tyr-Pro (409.1920 > 289.1800 *m/z*) were inserted as a small window for each chromatogram. The peak of the target molecule is indicated by an arrow. *N.D.* indicates not detected. The brain sample was also subjected to an APDS derivatization, followed by LC–MS/MS-SRM analysis as described in the Materials and methods section. (**c**) Time course of brain concentrations of Tyr-[^13^C_5_,^15^N]Pro after single oral administration at doses of 10 (solid line) and 100 mg/kg (dotted line) in mice. The values of each time point at the different doses were calculated using the present brain calibration curve. Each value is expressed as mean ± SEM (*n* = 3–5).

**Figure 5:**
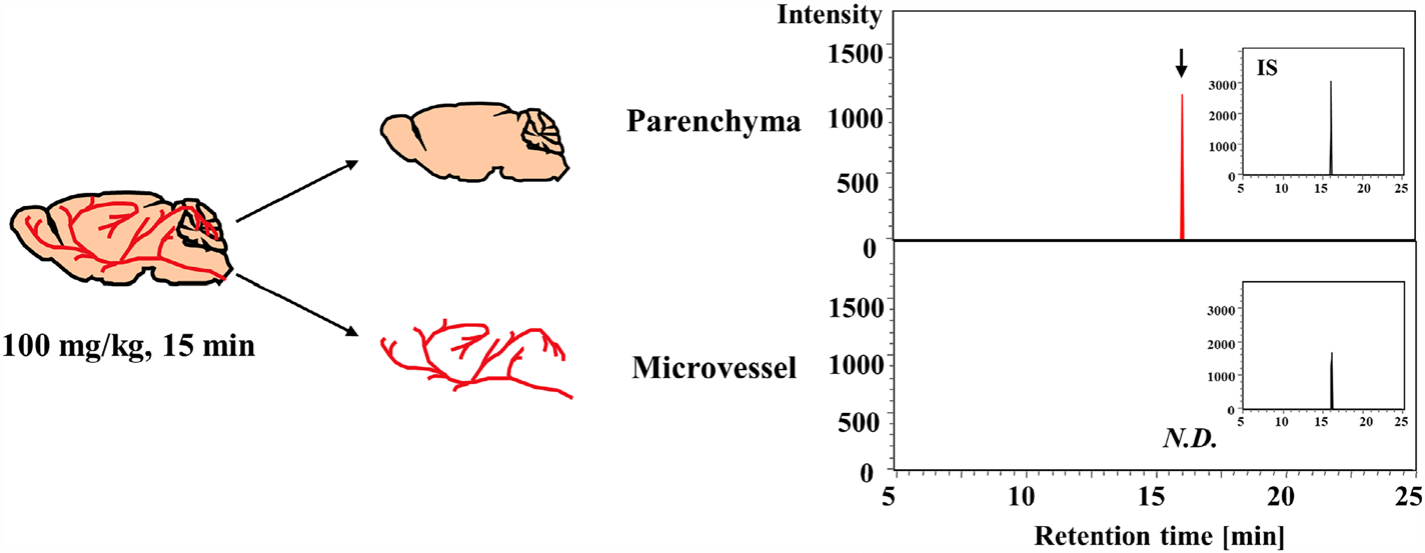
SRM chromatograms of LC–MS/MS analysis of APDS-Tyr-[^13^C_5_,^15^N]Pro in the parenchyma and microvessel fractions at 15 min after 100 mg/kg oral administration to ICR mice. Representative SRM chromatograms of APDS-Tyr-[^13^C_5_,^15^N]Pro (405.1800 > 285.1480 *m/z*) in the parenchyma and microvessel 15 min after a single oral administration of 100 mg/kg. The corresponding chromatograms of internal standard (IS) of [^13^C_9_,^15^N]Tyr-Pro (409.1920 > 289.1800 *m/z*) were inserted as a small window for each chromatogram. The peak of the target molecule was indicated by an arrow. *N.D.* indicates not detected. The brain sample was also subjected to an APDS derivatization, followed by LC–MS/MS-SRM analysis as described in the Materials and methods section.

We next aimed to confirm the entry of Tyr-[^13^C_5_,^15^N]Pro in brain tissue after oral administration at 10 and 100 mg/kg to ICR mice, using the present APDS-LC-qTOF/MS with a high sensitivity of > 6 fmol/mg-dry brain (LOD). As shown in Fig. 4a and b, significant MS detection of Tyr-[^13^C_5_,^15^N]Pro was observed 15 min after oral administration in both 10 and 100 mg/kg-dosed mouse brains. The *C*_max_ values of Tyr-[^13^C_5_,^15^N]Pro in the whole-brain tissue were 0.009 ± 0.004 and 0.089 ± 0.044 pmol/mg-dry brain, and *AUC*_0–120 min_ values were 0.34 ± 0.11 and 2.79 ± 1.25 pmol·min/mg-dry brain, for 10 and 100 mg/kg orally administrated doses, respectively (Fig. 4c and Table 2). Surprisingly, the *t*_max_ of Tyr-[^13^C_5_,^15^N]Pro in brain tissue was as rapid as 15 min, following which it rapidly decreased with time (*t*_1/2_, 16 and 12 min for 10 and 100 mg/kg, respectively) (Table 2). Notably, orally administered Tyr-[^13^C_5_,^15^N]Pro reached the mouse brain tissue without any degradation or in intact form; the percentage of Tyr-[^13^C_5_,^15^N]Pro accumulated in brain tissue (36.7 ± 11.9 pmol/whole brain at 10 mg/kg; 301.3 ± 135 pmol/whole brain at 100 mg/kg) was 2.5 ± 0.8% and 2.6 ± 1.2% following total absorption of Tyr-[^13^C_5_,^15^N]Pro in mouse plasma at doses of 10 (1.46 nmol/whole plasma) and 100 mg/kg (11.5 nmol/whole plasma), respectively. Figure 5 reveals that Tyr-[^13^C_5_,^15^N]Pro was accumulated in the brain parenchyma but not in brain microvessels, suggesting the observed brain accumulation (Fig. 4 and Table 2) was caused by intact transport across the BBB in mice.

**Table 2:** Pharmacokinetics parameters of Tyr-[^13^C_5_,^15^N]Pro in the brain after oral administration to ICR mice at 10 and 100 mg/kg.

Parameter	Dose (mg/kg)	
	10	100
*C*_max_ (pmol/mg-dry brain)	0.009 ± 0.004	0.089 ± 0.044
*t*_max_ (min)	15	15
*t*_1/2_ (min)	16	12
*AUC*_0–120 min_ (pmol·min/mg-dry brain)	0.34 ± 0.11	2.79 ± 1.25

*C*_max_ maximum brain concentration, *AUC*_0–120 min_ area under the brain concentration, *t*_max_ time to reach peak brain concentration, *t*_1/2_ elimination half-life.

Each value is expressed as mean ± SEM (*n* = 3–5).

### Local accumulation of Tyr-Pro in different brain regions of ICR mice

**Figure 6:**
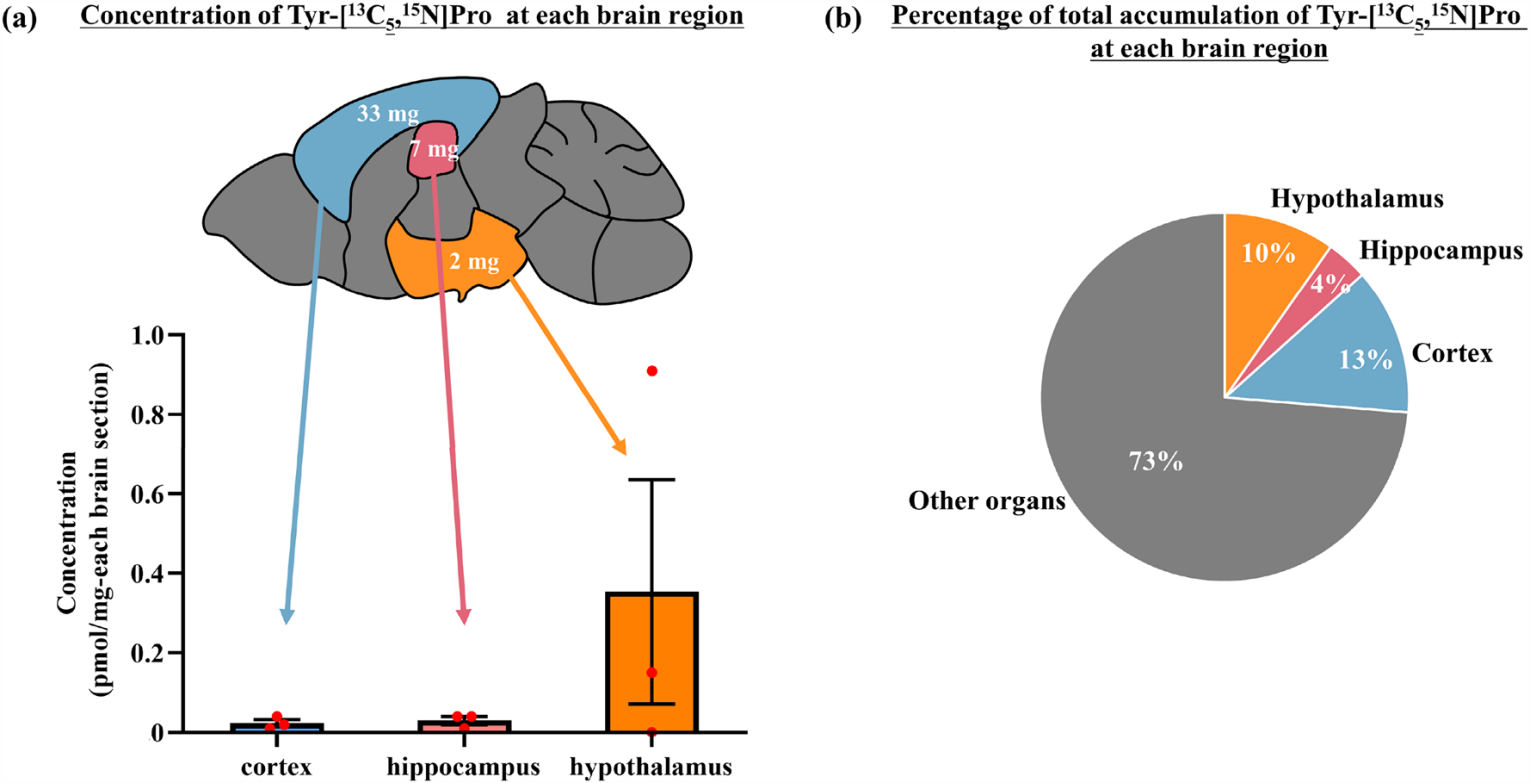
Accumulation of Tyr-[^13^C_5_,^15^N]Pro in different brain regions at 15 min after 100 mg/kg oral administration to ICR mice. (**a**) Distribution of the Tyr-[^13^C_5_,^15^N]Pro in different brain regions. The values were calculated using the brain calibration curve. Each value is expressed as mean ± SEM (*n* = 3). The weight of each brain region is shown in the brain tissue diagram in white letters. (**b**) Percentage of the total amount of Tyr-[^13^C_5_,^15^N]Pro in each brain region. The total amount of Tyr-[^13^C_5_,^15^N]Pro in each brain region was calculated according to the average contents multiplied by the tissue weight.

Given the preferable accumulation of Tyr-[^13^C_5_,^15^N]Pro in brain parenchyma of ICR mice following oral administration, we further examined local accumulation in relevant regions by APDS-LC-qTOF/MS, as we have previously reported that Tyr-Pro in mouse perfusion experiments can accumulate in the hippocampus, hypothalamus, cerebral cortex, striatum, and cerebellum^17^. Brain tissues at 15 min after oral administration of Tyr-Pro at 100 mg/kg were used for the local accumulation experiments, because of low LOD and limited weight of organ tissues. As shown in Fig. 6a, a significant accumulation of administered Tyr-[^13^C_5_,^15^N]Pro was detected in the hypothalamus (0.35 ± 0.28 pmol/mg-dry tissue), as well as in the hippocampus (0.03 ± 0.01 pmol/mg-dry tissue) and cortex regions (0.02 ± 0.01 pmol/mg-dry tissue) (Fig. 6), while no peak was observed in the control group (Fig. S1). Notably, the preferable local accumulation in the three regions accounted for 27% of the whole-brain accumulation (Fig. 6b).

## Discussion

Food-derived peptides are a class of molecules with multiple physiological functions in the tissues and fluids of living organisms^21^. To date, several peptides, such as PPKNW^22^, YPLDLF^23^, GYYPT^24^, VHVV^25^, MKP^26^ and NIPPLTQTPVVVPPFLQPE^27^, have been reported to potentially improve impaired memory and reduce the risk of age-related diseases such as AD. However, although these molecules have shown in vivo beneficial effects on brain function, their ability to reach the brain system in the intact form has not yet been discussed, given that peptides might undergo hydrolysis during absorption and restrict intact transport across the BBB^28–30^. Using the present APDS-aided LC-qTOF/MS, capable of highly sensitive detection of Tyr-Pro in brain tissue (> 6 fmol/mg-dry brain tissue), we aimed to establish the entry of intact Tyr-Pro, which has been shown to cross the BBB in a mouse perfusion study^17^, into the brain system after oral administration.

As shown in Fig. 4, we first report that orally administered Tyr-Pro or Tyr-[^13^C_5_,^15^N]Pro reached the brain in an intact form as early as 15 min post-administration. This finding strongly indicates that the memory-improving Tyr-Pro ^18^ stably crossed the intestinal membrane, was absorbed into blood circulation, and immediately reached the brain parenchyma of male mice (Figs. 4 and 5). However, considering the report that sex differences may affect the pharmacokinetics of target in mice^31^, further animal experiments using female mice must be needed to clarify the bioavailability of Tyr-Pro in mice. To the best of our knowledge, this is the first evidence highlighting the intact transport of peptides from the mouth to the brain, and it is noteworthy that peptides exhibit physiological potential as orally active memory-improving food compounds. The observed 15-min *t*_max_ of Tyr-[^13^C_5_,^15^N]Pro into ICR mouse blood circulation post-oral administration (Table 1) was rapid when compared with the reported absorption kinetics of dipeptides (e.g., *t*_max_ of Trp-His, 60 min^32^; *t*_max_ of Val-Tyr, 90 min^33^). The rapid absorption of Tyr-Pro into the blood system might be attributed to the high affinity with PepT1 transporter, similar to hydroxyPro-containing peptides^34^, and further kinetic studies concerning *K*_m_ and *V*_max_ using in vitro transport experiments with Caco-2 cell monolayers^35^ are ongoing. The kinetic studies will also elucidate the mechanism underlying the marked absorption magnitude of Tyr-Pro (*AUC*_0–120 min_, 1331 ± 267 pmol·min/mL-plasma at 10 mg/kg, Table 1) when compared with that of reported absorbable dipeptides, such as Trp-His (AUC_0-6 h_, 71.3 ± 18.7 pmol·h/mL-plasma, 10 mg/kg oral administration)^32^ and Val-Tyr (*AUC*_0–6 h_, 19.4 ± 0.71 pmol·h/mL-plasma, 30 mg/kg oral administration)^33^. The rapid accumulation of Tyr-[^13^C_5_,^15^N]Pro into the ICR mouse brain system 15 min after the oral administration (Table 2) could be explained by the rapid transport of the dipeptide at 0.5 min post-perfusion in a previous report^17^. Although the BBB transport routes, including L-type amino acid transporter 1 (LAT1) and peptide histidine transporter 2 (PHT2), for Tyr-Pro remain uncertain, the rapid transport of Tyr-Pro was comparable with that of drugs examined in mouse perfusion studies, including nicotine at 0.5 min^36^ and quinine at 0.25 min^37^. Acetaminophen has also been shown to rapidly accumulate in the brain as early as 15 min after oral administration at 100 mg/kg^38^, thereby strongly supporting the present result. According to the report by Ayoub et al*.*^39^ in which the penetration power (or brain/plasma accumulation ratio) of an anti-diabetic drug omarigliptin was 0.23 at 2 h, Tyr-Pro (0.036: 1.8 pmol/g-wet brain/49 pmol/mL-plasma at 15 min, Tables 1 and 2) may have a 1/6-fold lower penetration power compared to omarigliptin.

The transportability of compounds into the brain system is strictly regulated by the BBB^40^. Currently, no report has established the entry of bioactive peptides across the BBB after “oral” administration. Herein, we demonstrated a significant accumulation of Tyr-[^13^C_5_,^15^N]Pro in the mouse brain parenchyma with *AUC*_0–120 min_ of 0.34 ± 0.11 and 2.79 ± 1.25 pmol·min/mg-dry brain at oral doses of 10 and 100 mg/kg, respectively (Table 2). The accumulation of Tyr-Pro (0.0037% at 10 mg/kg and 0.0027% at 100 mg/kg from oral dose) was markedly low to achieve effective acute action, compared with drug compounds. Courad et al. have reported that acetaminophen, known to exhibit an analgesic effect via the central nervous system (CNS), reached a brain accumulation of ~ 0.1% from oral dose of 100 mg/kg^38^. Another report claimed morphine brain accumulation of ~ 0.01% from oral dose of 9 mg/kg^41^. Considering a brain-transportable natural compound, 1-deoxynojirimycin showed an appropriate accumulation of ~ 1.2 pmol/mg in the brain of Sprague–Dawley rats after oral administration at 40 μmol or 6.5 mg/kg^42,43^. Conversely, despite the low accumulation or poor uptake ratio of 0.0037% of Tyr-[^13^C_5_,^15^N]Pro in the mouse brain at 10 mg/kg observed in the present oral administration study, we have previously shown significant improvement in impaired memory in amyloid β-induced AD mice following daily Tyr-Pro intake (100 mg/kg, twice a day) for 16 days^18^ suggesting that low Tyr-Pro accumulation, even at 100 mg/kg, would be effective when one expects a preventive effect against the onset of AD.

In the present study, we demonstrated the preferable accumulation of orally administrated Tyr-Pro in the cortex, hippocampus, and hypothalamus (Fig. 6 and Fig. S1). The regions of Tyr-Pro accumulation were in good agreement with the regions visualized by MALDI-MS imaging in mouse perfusion experiments of Tyr-Pro^17^. The hypothalamus regulates body temperature and sleep patterns, controls hunger and thirst, and plays a role in memory and emotional actions, while the hippocampus and cortex may be involved in memory, learning, navigation, and perception of space^44^. Thus, as speculated based on previous reports on the brain health benefits of Tyr-Pro by local accumulation in the aforementioned regions^17,18^, a long-term administration study of Tyr-Pro is currently in progress.

The preventive mechanism of AD and the effective dose of Tyr-Pro for oral intake remain unclear. To date, acetylcholinesterase (AChE) inhibitors, such as donepezil^45^, have been developed to suppress the reduction of the neurotransmitter factor ACh^45^. In contrast, Tyr-Pro accumulation in the brain reportedly induces upregulation of choline acetyltransferase (ChAT) expression^18^, thereby promoting ACh production. Although the action of Tyr-Pro in the brain differs from that of the drug, regulation of the cholinergic nervous system is an appropriate target for AD prevention via peptide intake. The mechanism underlying the upstream signaling cascade for ChAT activation via Tyr-Pro is currently under investigation using NE-4C neural stem cell-line experiments.

In conclusion, we provided a novel finding that intact Tyr-Pro can reach the brain after oral administration to ICR mice. The detection of Tyr-Pro, i.e., 15 min after oral administration, strongly indicates that the transport from the blood to the brain across the BBB was rapid following entry into the blood circulation. The APDS derivatization-aided LC-qTOF/MS method also demonstrated the accumulation of Tyr-Pro in brain tissues at > 0.02 pmol/mg-dry brain, in particular, in the cortex, hippocampus, and hypothalamus, with the transport ratio of 2.6% of total absorption in blood circulation, and 0.0027% of 100 mg/kg administered dose.

## Materials and methods

### Chemicals and reagents

Tyr-[^13^C_5_,^15^N]Pro (Lot: SQ21192, SQ23862 and SQ25025) and [^13^C_9_,^15^N]Tyr-Pro (Lot: SQ25407) were synthesized by Scrum Co. (Tokyo, Japan). APDS (Lot: EFJ0223, DGG0331) was purchased from FUJIFILM Wako Co. (Osaka, Japan). Distilled water, methanol (MeOH), acetonitrile (ACN) and formic acid (FA) were of LC–MS grade (Merck, Darmstadt, Germany). All other reagents used in the present study were of analytical grade and used without further purification.

### Animal experiments

The present study was performed using seven- to nine-week-old male ICR mice weighing 25–35 g (Jcl:ICR; CLEA Japan, Tokyo, Japan). All mice were housed for one week under controlled temperature (21 ± 1 °C), humidity (55 ± 5%), and a 12 h-light–dark cycle from 8:00 am to 8:00 pm. The mice were fed a laboratory diet (CE-2, CLEA Japan) and provided water ad libitum. The mice were fasted for 16 h prior to the oral administration of Tyr-[^13^C_5_,^15^N]Pro. All animal experiments were conducted in accordance with the Proper Conduct of Animal Experiments and Related Activities in Academic Research Institutions under the jurisdiction of the Ministry of Education, Culture, Sports, Science, and Technology in Japan. All methods were reported in accordance with ARRIVE guidelines. The Ethics Committee on Animal Experiments at Fukuoka University approved all the experimental protocols (permit number: 1915137).

### Administration experiments

On the experimentation day, mice were randomly divided into three groups: (1) control group, (2) 10 mg/kg Tyr-[^13^C_5_,^15^N]Pro group, and (3) 100 mg/kg Tyr-[^13^C_5_,^15^N]Pro group. The control group was administered water, and the other two groups were administered 10 or 100 mg/kg of Tyr-[^13^C_5_,^15^N]Pro dissolved in water. Blood and brain were collected from each mouse at 15, 30, 60, and 120 min post-administration (*n* = 3–5 at each scheduled time). Blood was collected from the left ventricle of the heart under anesthesia with 40% urethane (Sigma-Aldrich, St. Louis, MO, USA) and placed into a 1.5 mL tube containing ethylenediaminetetraacetic acid disodium salt (EDTA-2Na, ~ 5 mg/tube). The whole brain was harvested according to the following procedure: Briefly, after the descending thoracic aorta was ligated, the left jugular aorta was sectioned and perfused with 10 mL of freshly prepared physiological buffer (141 mM NaCl, 4 mM KCl, 2.8 mM CaCl_2_, 1 mM MgSO_4_∙7H_2_O, 1 mM NaH_2_PO_4_∙2H_2_O, 10 mM d-glucose, and 10 mM HEPES, pH 7.4) to wash out any blood from the brain. After the perfusion with a physiological buffer, the whole brain was removed from the mice by decapitation and subsequently weighed. Blood samples were then centrifuged at 3500 × *g* at 4 °C for 15 min to obtain a plasma specimen, followed by storage at -30 °C until LC-qTOF/MS analysis. Brain samples (wet weight, 0.5 g/mouse) were immediately frozen in liquid nitrogen following lyophilization. Lyophilized brain samples (dry weight, 0.1 g/mouse) were stored at − 30 °C until analysis. To determine Tyr-[^13^C_5_,^15^N]Pro in local regions of the brain, a separate administration study was performed, examining the hypothalamus (1.6 ± 0.2 mg), hippocampus (7.3 ± 0.2 mg), cortex (33.4 ± 0.3 mg), and other organs (58.5 ± 2.0 mg) of three mice at 15 min post-administration of 100 mg/kg Tyr-[^13^C_5_,^15^N]Pro. After lyophilization, the samples were stored at − 30 °C until analysis.

### Preparation of brain parenchyma and microvessel fractions

The parenchyma and microvessel fractions were separated from the mouse brain 15 min after Tyr-[^13^C_5_,^15^N]Pro administration (100 mg/kg), according to previous reports^17,46^. In a separate administration experiment, brain samples from a mouse were minced using a glass homogenizer in physiological buffer (0.5 mL), followed by further homogenization after adding 1.0 mL of 26.5% dextran at 4 °C. The homogenate obtained was centrifuged at 5400 × *g* for 15 min at 4 °C. The supernatant, representing the parenchymal fraction, and the pellet, representing the microvessel fraction, were carefully collected. Subsequently, 1 mL of physiological buffer was added to the supernatant and centrifuged at 5400 × *g* for 15 min at 4 °C to obtain the brain parenchyma. Considering the pellet, 1.5 mL of 17.7% dextran was added to the pellet, followed by centrifugation at 5400 × *g* for 15 min at 4 °C to obtain the microvessel fraction. Both parenchyma and microvessel fractions were frozen and stored at − 30 °C until analysis.

### LC-qTOF/MS analysis combined with APDS derivatization technique for determining Tyr-[^13^C_5_,^15^N]Pro in plasma

To determine plasma Tyr-[^13^C_5_,^15^N]Pro, a plasma aliquot (10 μL) was mixed with 180 μL of 0.1% Triton X-100 solution containing 0.1% FA, following the addition of 10 μL of 0.1 μmol/L [^13^C_9_,^15^N]Tyr-Pro as IS. The solution was thoroughly mixed by vortexing and loaded onto a Sep-Pak Vac C18 cartridge (Waters Co., Milford, MA, USA), which was pre-conditioned sequentially with 1 mL of MeOH containing 0.1% FA and 1 mL of 0.1% FA. The cartridge was washed with 2 mL 0.1% FA and eluted with 2 mL 30% MeOH containing 0.1% FA at 1.0 mL/min. The eluent was evaporated to dryness and dissolved in 60 μL of borate buffer (100 mM, pH 10). After adding 40 μL of APDS solution (300 mM in ACN), the APDS derivatization reaction was initiated for 20 min at 60 °C. Thereafter, the APDS reaction was stopped by adding 100 µL of 0.1% FA solution, and an aliquot (100 µL) of the solution was filtered with a Millex-LG syringe filter (Millipore Co., Carrigtwohill, Ireland) and subjected to LC-qTOF/MS analysis. A calibration curve of Tyr-[^13^C_5_,^15^N]Pro in mouse plasma was obtained by spiking 10 µL of different concentrations of Tyr-[^13^C_5_,^15^N]Pro (at final concentrations of 10–1500 pmol/mL-plasma), along with 10 µL of 0.1 μmol/L [^13^C_9_,^15^N]Tyr-Pro (100 pmol/mL-plasma) as IS, into 10 µL of blank plasma. The MS intensity ratio of the observed peak area of the peptide to that of the IS was used to establish the calibration curve. The LOD and LOQ of Tyr-[^13^C_5_,^15^N]Pro were determined as 3.3 × standard deviation (SD)/slope and 10 × SD/slope, respectively.

The LC system comprised an Agilent 1200 series HPLC system (Agilent Technologies, Waldbronn, Germany), and LC separation was conducted on a 5C18-AR-II (2.0 I.D. × 150 mm, particle size, 5 μm, Nacalai Tesque Co. Kyoto, Japan) with a gradient elution at 40 °C (flow rate: 0.25 mL/min; A solution: 0.1% FA in water; B solution: 0.1% FA in MeOH; gradient condition: 0–100% B solution in 20 min). qTOF/MS analysis was performed using Compact (Bruker Daltonics, Bremen, Germany), in the electrospray ionization (ESI)-positive ion mode. The SRM measurements were set as follows: drying N_2_ gas, 8.0 L/min; drying temperature, 200 °C; nebulizer pressure, 2.0 bar; capillary, 4500 V; end plate offset, 500 V; mass range, 50–1000 *m/z*. The monoisotopic isolations (*m/z*) at a molecular ion selection width of 3 *m/z* were 405.1800 > 285.1480 and 409.1920 > 289.1600 for Tyr-[^13^C_5_,^15^N]Pro and [^13^C_9_,^15^N]Tyr-Pro, respectively. The cone voltage was set to 27 V, and the collision energies were set to 17 and 25 eV for Tyr-[^13^C_5_,^15^N]Pro and [^13^C_9_,^15^N]Tyr-Pro, respectively. An MS calibration solution (10 mM sodium formate in 50% ACN) was injected at the beginning of each run, and all spectra were calibrated internally. MS data were analyzed using Bruker Data Analysis software (ver. 3.2). All SRM chromatograms had a width of 0.005 *m/z*.

### LC-qTOF/MS analysis combined with APDS derivatization technique for determining Tyr-[^13^C_5_,^15^N]Pro in the brain

A lyophilized brain sample was pounded using the BioMasher II (Nippi. Inc, Tokyo, Japan), and 2.9 mL of 0.1% Triton X-100 solution containing 0.1% FA and 0.1 mL of 30 nmol/L [^13^C_9_,^15^N]Tyr-Pro as IS (0.1 pmol/mg-dry brain) was added to an aliquot (~ 30 mg) of the brain powder. The solution was then homogenized using a Polytron homogenizer (Kinematica AG, Luzern, Switzerland) at 20,000 rpm for 60 s twice at 4 °C, followed by sonication using the SONIFIRE 250 (Branson Ultrasonics, Emerson Japan Co., Kanagawa, Japan) with an output control of 3 for 10 s, performed three times at 4 °C. The prepared samples were centrifuged at 7000 × *g* for 15 min at 4 °C. The supernatant was subjected to a Sep-Pak Vac C18 cartridge (Waters Co.), followed by APDS derivatization and LC-qTOF/MS analysis. Briefly, the cartridge was pre-conditioned sequentially with 3 mL of MeOH containing 0.1% FA and 3 mL of 0.1% FA, washed with 5 mL of 0.1% FA and then eluted with 3 mL of 30% MeOH containing 0.1% FA at 1.0 mL/min. The eluent was evaporated to dryness and then applied to APDS derivatization as described in the aforementioned protocols. A calibration curve of Tyr-[^13^C_5_,^15^N]Pro in mouse brain was obtained by spiking 100 µL of different concentration of Tyr-[^13^C_5_,^15^N]Pro (at final concentrations of 0.005–0.075 pmol/mg-dry brain, respectively), along with 100 µL of 30 nmol/L [^13^C_9_,^15^N]Tyr-Pro (0.1 pmol/mg-dry brain) as IS, into 30 mg blank brain homogenate.

### Data analysis

The concentration–time pharmacokinetic data were analyzed using GraphPad Prism software (ver. 9.4.0, GraphPad; La Jolla, CA). *C*_max_, *t*_max_, and *AUC*_0–120 min_ values were obtained by measuring the accumulated target substance after each observation period. The *t*_1/2_ value was obtained using the equation *t*_1__/2_ = 0.693/k^32^. The elimination rate constant (k) was determined by linear regression analysis of data points plotted between *t*_max_ and 60 min. The absorption ratio of Tyr-Pro in plasma or brain was obtained by dividing *AUC*_0–120 min_ in whole plasma volume (1.1 mL/mouse) or *AUC*_0–120 min_ in whole dry brain weight (108 mg/mouse) by oral dose, respectively. Results are expressed as the mean ± standard error of the mean (SEM).

### Supplementary Information

Supplementary Information.

## Data Availability

All data generated or analyzed during this study are included in this article (and its supplementary files).
