# Supplementary material for: A memory-improving dipeptide, Tyr-Pro, can reach the mouse brain after oral administration
Source: Sci Rep. 2023 Oct 7;13:16908. doi: 10.1038/s41598-023-44161-z (PMC10560274; doi:10.1038/s41598-023-44161-z)
Supplement: Supplementary file 1 — Supplementary Information. [file 41598_2023_44161_MOESM1_ESM.pdf]

Supplemental information

A memory-improving dipeptide, Tyr-Pro, can reach the mouse brain after oral administration

Lihong Cheng[1#], Mitsuru Tanaka[1#], Atsuko Yoshino[1], Yuki Nagasato[1], Fuyuko Takata[2], Shinya Dohgu[2], Toshiro Matsui[1,*]

[1]Department of Bioscience and Biotechnology, Faculty of Agriculture, Graduate School of Kyushu University, Japan

[2]Faculty of Pharmaceutical Sciences, Fukuoka University, Japan

[*]**Corresponding author**: Toshiro Matsui, Ph.D., Department of Bioscience and Biotechnology, Faculty of Agriculture, Graduate School of Kyushu University, 744 Motooka, Nishi-ku, Fukuoka 819-0395, Japan

Email: tmatsui@agr.kyushu-u.ac.jp

Tel/Fax: +81-92-802-4752

[#]Lihong Cheng and Mitsuru Tanaka contributed equally to this work.

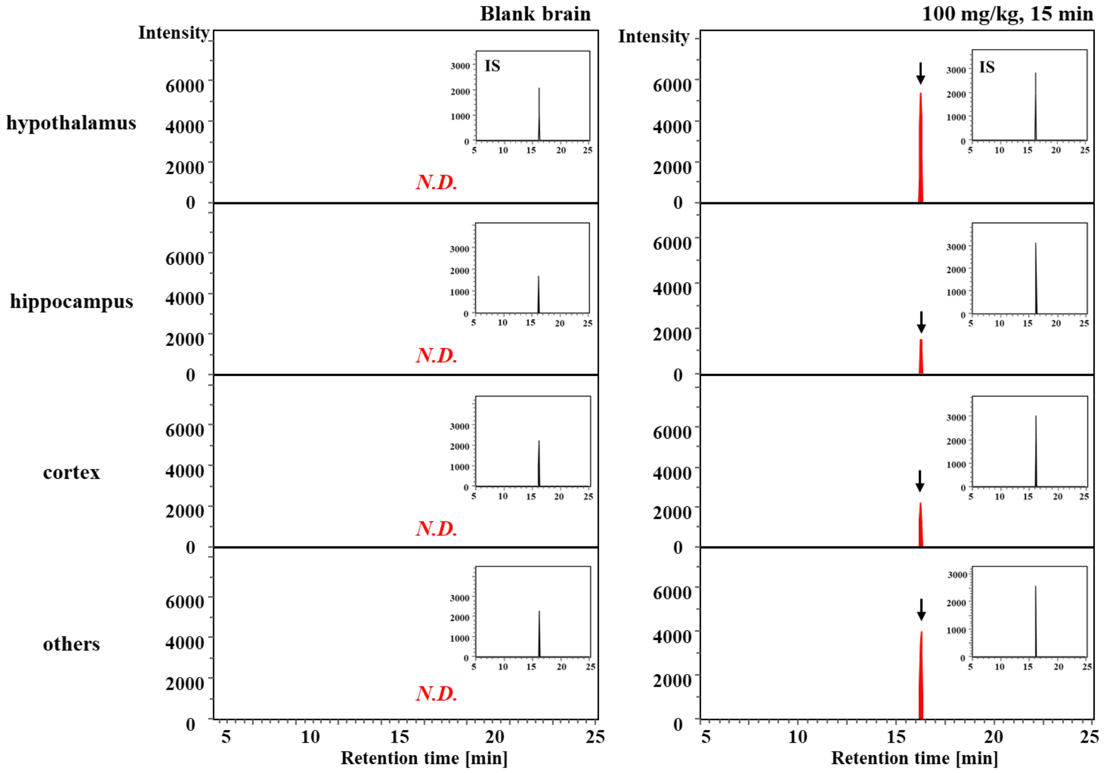

Figure S1. SRM chromatograms of LC-MS/MS analysis of APDS-Tyr-[$^{13}C_5$,^{15}N]Pro in different brain regions at 15 min after 100 mg/kg oral administration. Representative SRM chromatograms of APDS-Tyr-[$^{13}C_5$,^{15}N]Pro (405.1800 > 285.1480 *m/z*) in different brain regions with or without (blank) single 100 mg/kg oral administration after 15 min. The corresponding chromatograms of internal standard (IS) of [$^{13}C_9$,^{15}N]Tyr-Pro (409.1920 > 289.1800 *m/z*) were inserted as a small window for each chromatogram. The peak of the target molecule is indicated by an arrow. *N.D.* indicates not detected. Each sample was subjected to an APDS derivatization, followed by SRM-LC-qTOF/MS analysis as described in the Materials and Methods section.

Table S1 SRM parameters for LC-MS/MS analysis.

Analyte	RT (min)	Precursor ion	Precursor (*m/z*)	Product (*m/z*)	Cone voltage (V)	CE (eV)
APDS-Tyr-[$^{13}C_5$, ^{14}N]Pro	16.2	$[M+H]^+$	405.1800	285.1480	27	17
APDS-[$^{13}C_9$,^{15}N]Tyr-Pro	16.2	$[M+H]^+$	409.1920	289.1600	27	25

RT : chromatographic retention times (RT), CE : collision energies for each analyte.

Table S2 Parameters of standard curve making in plasma and brain.

Parameter	Equation	Range	R^2	LOD	LOQ
Plasma	y = 0.0295x + 0.0101	10–1500 pmol/mL-plasma	0.9891	9.99 pmol/mL-plasma	30.3 pmol/mL-plasma
Brain	y = 37.363x + 0.0706	0.005–0.075 pmol/mg-dry brain	0.9954	0.006 pmol/mg-dry brain	0.019 pmol/mg-dry brain

In each equation, y is the ratio of the observed peak area to that of the internal standard (IS) and x is the concentration (pmol/mL-plasma) in the plasma and (pmol/mg-dry brain) brain. The limit of detection (LOD) and quantification (LOQ) of the analytical method were determined based on a linear regression analysis of the mean values of the calibration standards (n = 3). The LOD and LOQ were determined as follows: LOD = 3.3 × SD/slope and LOQ = 10 × SD/slope, where SD is the standard deviation of the y-intercept from the regression analysis.
